# Therapeutic Potential of a Novel *Bifidobacterium* Identified Through Microbiome Profiling of RA Patients With Different RF Levels

**DOI:** 10.3389/fimmu.2021.736196

**Published:** 2021-11-15

**Authors:** Yunju Jeong, JooYeon Jhun, Seon-Yeong Lee, Hyun Sik Na, JeongWon Choi, Keun-Hyung Cho, Seung Yoon Lee, A Ram Lee, Sang-Jun Park, Hyun Ju You, Ji-Won Kim, Myeong Soo Park, Bin Kwon, Mi-La Cho, Geun Eog Ji, Sung-Hwan Park

**Affiliations:** ^1^ Department of Food and Nutrition, Research Institute of Human Ecology, Seoul National University, Seoul, South Korea; ^2^ Research Center, BIFIDO Co., Ltd., Hongcheon, South Korea; ^3^ Rheumatism Research Center, Catholic Research Institute of Medical Science, College of Medicine, The Catholic University of Korea, Seoul, South Korea; ^4^ Lab of Translational ImmunoMedicine, Catholic Research Institute of Medical Science, College of Medicine, The Catholic University of Korea, Seoul, South Korea; ^5^ Department of Biomedicine & Health Sciences, College of Medicine, The Catholic University of Korea, Seoul, South Korea; ^6^ Institute of Environmental Health, School of Public Health, Seoul National University, Seoul, South Korea; ^7^ N-Bio, Seoul National University, Seoul, South Korea; ^8^ Division of Rheumatology, Department of Internal Medicine, Daegu Catholic University School of Medicine, Daegu, South Korea; ^9^ Department of Medical Life Science, College of Medicine, The Catholic University of Korea, Seoul, South Korea; ^10^ Division of Rheumatology, Department of Internal Medicine, Seoul St. Mary’s Hospital, College of Medicine, The Catholic University of Korea, Seoul, South Korea

**Keywords:** rheumatoid arthritis, rheumatoid factor, microbiome, *Bifidobacterium longum*, T helper 17 cell

## Abstract

The potential therapeutic effects of probiotic bacteria in rheumatoid arthritis (RA) remain controversial. Thus, this study aimed to discover potential therapeutic bacteria based on the relationship between the gut microbiome and rheumatoid factor (RF) in RA. Bacterial genomic DNA was extracted from the fecal samples of 93 RA patients and 16 healthy subjects. Microbiota profiling was conducted through 16S rRNA sequencing and bioinformatics analyses. The effects of *Bifidobacterium* strains on human peripheral blood mononuclear cells and collagen-induced arthritis (CIA) mice were assessed. Significant differences in gut microbiota composition were observed in patients with different RF levels. The relative abundance of *Bifidobacterium* and *Collinsella* was lower in RF-high than in RF-low and RF-negative RA patients, while the relative abundance of *Clostridium* of Ruminococcaceae family was higher in RF-high than in RF-low and RF-negative patients. Among 10 differentially abundant *Bifidobacterium*, *B. longum* RAPO exhibited the strongest ability to inhibit IL-17 secretion. Oral administration of *B. longum* RAPO in CIA mice, obese CIA, and humanized avatar model significantly reduced RA incidence, arthritis score, inflammation, bone damage, cartilage damage, Th17 cells, and inflammatory cytokine secretion. Additionally, *B. longum* RAPO significantly inhibited Th17 cells and Th17-related genes—*IL-17A*, *IRF4*, *RORC*, *IL-21*, and *IL-23R*—in the PBMCs of rheumatoid arthritis patients. Our findings suggest that *B. longum* RAPO may alleviate RA by inhibiting the production of IL-17 and other proinflammatory mediators. The safety and efficacy of *B. longum* RAPO in patients with RA and other autoimmune disorders merit further investigation.

## Introduction

Rheumatoid factor (RF) is an autoantibody detected in approximately 80% of patients with the autoimmune disease rheumatoid arthritis (RA) (1, 2). In addition to RA, RF has been implicated in other autoimmune diseases, including Sjögren’s syndrome and systemic lupus erythematosus (3, 4). The presence of RF has also been reported in older individuals; however, its role remains unclear (5). Additionally, the mechanisms contributing to variation in RF levels in patients with RA are poorly understood (6–8).

Mounting evidence suggests the critical role of human RF as an IgG antigen in RA development and pathogenesis (9). Additionally, antigens of infectious microorganisms, such as Epstein–Barr virus, cytomegalovirus, parvovirus B19, and *Porphyromonas gingivalis*, can trigger RA development (10–14). The deposition of immune complexes containing RF in the joints activates the complement, resulting in type III hypersensitivity reactions and causing joint inflammation (15). During this inflammatory reaction, the membrane attack complex consisting of C5a and C5b-C9 is formed due to complement activation (15). C5a induces the migration of neutrophils and polymorphonuclear neutrophils to synovial cavities, and C5b-C9 activates peptidyl arginine deaminating enzymes (PADs) in neutrophils by attacking their cell membranes (9). Hypercitrullinated proteins activated by PADs and non-resolving inflammation increase the antigen pool in RA (9). A recent clinical study has shown that the presence of RF was linked to RA severity (16). Furthermore, RF has been identified as a predictor of drug response in patients with RA treated with anti-TNFα drugs (7).

The gut microbiome has emerged as a key regulator of RA development and progression. Notably, commensal segmented filamentous bacteria have been reported to activate T helper (Th)-17 lymphocytes in the lamina propria, inducing autoantibody production and aggravating RA (17). In a mouse RA model, Maeda et al. identified gut dysbiosis as an RA risk factor, as it activates autoreactive T cells and Th17 cells in the large intestine (18). Human studies have confirmed the strong association between gut microbiota and RA. Staggering differences in the diversity and composition of the gut microbiome have been reported in patients with new-onset RA (NORA). In particular, Scher et al. reported that the relative abundance of *Prevotella copri* was higher in patients with NORA than in healthy subjects, suggesting *P. copri* as a potential pathogen driving RA (19). Furthermore, a 27-kDa protein of *P. copri* has been shown to induce potent immune responses by stimulating Th1 cells in SKG mice (20). Meanwhile, the higher abundance of Oxalobacteraceae family showed a protective effect on RA in a large-scale association analysis with 24 population-based cohorts (21).

Notwithstanding the increasing evidence of the role of microbiome in RA, the relationship between the gut microbiome and RF is understudied. In this study, we conducted microbiome analyses using bioinformatics to investigate the association between intestinal microflora and RF levels. We also performed *in vivo* studies to determine whether a single strain of *Bifidobacterium*, which was found based on the microbiome analysis, could modulate RA symptoms in an RA mouse model.

## Material and Methods

### Inclusion and Exclusion Criteria

In this study, we enrolled 93 RA patients and 16 healthy subjects (**Table 1**). Patients with RA fulfilled the 2010 American College of Rheumatology and European League Against Rheumatism classification criteria (22). RA patients were classified based on RF levels into RF-negative (*n* = 16), RF-low (*n* = 24), and RF-high (*n* = 53) groups. Patients with RF ≤20 IU/ml were considered RF-negative, those with RF levels between 20 and 60 IU/ml were considered RF-low, and those with RF >60 IU/ml were considered RF-high. Subjects who had recently used antibiotics, probiotics, or prebiotics were excluded from the study. The demographic and clinical characteristics of the study subjects are summarized in **Table 1**. The age of healthy subjects (37–62) and RA patients including RF-negative (41–57), RF-low (40–59), and RF-high (40–60) was not significantly different (**Table 1**). The ratio of female among the subjects is 94% from healthy control subjects, 100% from RF-negative, 92% from RF-low, and 85% from RF-high. Additionally, disease activity score in 28 joints (DAS28), erythrocyte sedimentation rate (ESR), C-reactive protein levels, and current use of methotrexate or a biological disease-modifying anti-rheumatic drug were not statistically different between RF-negative, RF-low, and RF-high RA patients. The study design was approved by the Institutional Review Board of Seoul St. Mary’s Hospital, The Catholic University of Korea (approval ID: KC17TNSI0570). Written informed consent was obtained from all study participants.

**Table 1:** Clinical features of research subjects.

	RA patients				Control
	Positive			Negative (NG) (RF ≤ 20)	Normal (NM)
	Low positive (LP) (20 < RF ≤ 60)	High positive (HP) (RF > 60)	Total		
**N**	24	53	77	16	16
**Age**	51.1 ± 5.9	51.2 ± 5.7	51.2 ± 5.7	50.9 ± 5.5	49.2 ± 9.6
**Female**	22 (92)	45 (85)	67 (87)	16 (100)	15 (94)
**DMARD-naive**	5 (21)	16 (30)	21 (27)	3 (19)	–
**Current use of MTX**	19 (79)	33 (62)	52 (68)	12 (75)	–
**Current use of bDMARD**	9 (38)	15 (28)	24 (31)	6 (38)	
RF**** (IU/ml)	34.8 ± 10.4	279.5 ± 230.9	203.2 ± 222.5	8.4 ± 5.9	–
**Anti-CCP (%, NG/LP/HP)**	29.2/0/70.8	18.9/7.6/73.6	22.08/5.19/72.73	25.0/12.5/62.5	
**DAS28 (score)**	2.4 ± 0.8	2.8 ± 1.3	2.63 ± 1.16	2.8 ± 1.5	–
**ESR (mm/h)**	12.7 ± 8.6	16.1 ± 15.6	15.0 ± 13.8	12.9 ± 8.3	–
**CRP (mg/dl)**	0.3 ± 0.5	0.6 ± 1.6	0.5 ± 1.3	0.4 ± 0.7	–

All values are presented as mean ± standard deviation, n (%), or n. All statistical analyses were done with Kruskal–Wallis test and chi-square test. The quadruple asterisks indicate p-value under 0.0001.

DMARD, disease-modifying anti-rheumatic drug; MTX, methotrexate; bDMARD, biological DMARD.

### Microbiota Analysis

Individual human fecal samples were sent immediately with ice to the research site after collection in a plastic container and were stored at −70°C within 12 h of arrival. Isolation of the bacterial genomic DNA from the fecal samples was performed using a QIAamp DNA Stool Mini Kit (Qiagen, Hilden, Germany) following the instructions of the manufacturer, followed by bead beating on a TissueLyser system (Qiagen). Quantification of the bacterial genomic DNA was carried out using a Qubit 3.0 Fluorometer (Thermo Fisher Scientific, Waltham, MA, USA). For sequencing, 16S rRNA gene amplification and index PCR were conducted following the Illumina 16S Metagenomic Sequencing Library preparation guide (Illumina, San Diego, CA, USA). For amplification of V3 and V4 regions, the 16S sequence was amplified using forward primer (5′-TCGTCGGCAGCGTCAGATGTGTATAAGAGACAGCCTACGGGNGGCWGCAG-3′) and reverse primer (5′-GTCTCGTGGGCTCGGAGATGTGTATAAGAGACAGGACTACHVGGGTATCTAATCC-3′). For indexing, Nextera XT Index 1 and 2 Primers from a Nextera XT Index kit (Illumina) were used. Each PCR product was purified using AMPure XP beads (Beckman Coulter, Pasadena, CA, USA). DNA sequencing was conducted in using the paired-end method (300 bpx2) with an Illumina MiSeq instrument according to the Illumina protocol. Raw 16S rRNA sequences were bioinformatically analyzed using QIIME 2 version 2019.4 (23), as previously described (24).

### Bacteria Isolation From Human Fecal Samples

Transgalactooligosaccharide (TOS) antibiotic mupirocin agar medium, a selective medium for *Bifidobacterium*, was used to separate *Bifidobacterium* from 100 mg of human fecal samples. After plating fecal samples diluted with phosphate-buffered saline (PBS) and culturing for 30 h at 37°C in an anaerobic jar under anaerobic condition, colonies were randomly taken from a plate from each sample and inoculated with cysteine De Man, Rogosa, and Sharpe (cMRS) broth and cultured for 24 h. The above process was performed in a glove box for blocking oxygen. Identification of bacteria strain was done with 16S rRNA sequencing service of Macrogen Inc. (Seoul, Korea).

### Bacterial Preparation

Bacterial samples (50µl) were inoculated into 5 L of cMRS liquid medium and incubated for 20 h at 37°C. After incubation, the bacteria were collected *via* centrifugation (2236HR high-speed centrifuge; Gyrozen, Gimpo, Korea) at 20°C and 7,000 rpm. After two washes with PBS, the bacterial samples were dried at 36°C and centrifuged at 2,000 rpm for 24 h using a Scanvac Speed Vacuum Concentrator (LaboGene Aps, Lillerød, Denmark). All bacteria used in this study were isolated from healthy subjects and RA patients. Donor characteristics are summarized in **Table 1**.

### Mice

Seven-week-old male DBA/1J mice (Orient Bio, Gyeonggi-do, Korea) and 7-week-old male NOD-scid IL2rγ^null^ (NSG) mice (Jackson Laboratory, Bar Harbor, ME, USA) were maintained under specific pathogen-free conditions in an animal facility with controlled humidity (55 ± 5%), light (12 h/12 h light/dark), and temperature (22 ± 1°C). The air in the facility passed through a HEPA filter system designed to exclude bacteria and viruses. Animals were fed mice chow (normal chow and 60 kcal chow) and water *ad libitum*. All experimental procedures were approved by the Institutional Animal Care and Use Committee at the School of Medicine and the Animal Research Ethics Committee of the Catholic University of Korea and were conducted in accordance with the Laboratory Animals Welfare Act according to the Guide for the Care and Use of Laboratory Animals. All experimental procedures were evaluated and conducted in accordance with the protocols approved by the Animal Research Ethics Committee at the Catholic University of Korea (Permit Number: CUMC 2019-0242-01). All procedures performed in this study followed the ethical guidelines for animal use.

### Generation of Collagen-Induced Arthritis and Obese Collagen-Induced Arthritis Mice

Collagen-induced arthritis (CIA) was established in male DBA/1J mice. Briefly, mice were inoculated with 100 µg of chicken type II collagen (CII; Chondrex Inc., Redmond, WA, USA) liquified overnight in 0.1 N acetic acid (4 mg/ml) in complete Freund’s adjuvant. Immunizations were performed intradermally *via* injection into the bottom of the tail. At 2 weeks following immunization, the mice were boosted with 100 µg of CII in incomplete Freund’s adjuvant (Chondrex Inc.). In addition, the obese CIA mouse group was fed a high-fat diet (60 kcal of fat) at primary immunization. A HFD was maintained until the sacrifice.

### Generation of Humanized Avatar Arthritis Model

To induce arthritis in humanized mice, 5 × 10^5^ peripheral blood mononuclear cells (PBMCs) of RA patients were injected into the tail vein. At 2 weeks after, orbital blood was collected to confirm engraftment by flow cytometry. Orbital blood was immunostained with CD4 T cells and CD8 T cells, and the cells were stained with Alexa Flour 700-conjugated anti-human CD4 antibody (BD Biosciences, San Diego, CA, USA) and allophycocyanin (APC)-Cy7-conjugated anti-human CD8 (BD Biosciences). Thereafter, 1 week after confirmation of engraftment, the mice were sensitized with 50 µg of lipopolysaccharide (Sigma Aldrich, St. Louis, MO, USA). Each *in vivo* experiment was repeated a total of three times.

### Clinical Assessment of Arthritis

Arthritis severity was determined using the mean arthritis index, which ranges from 0 to 4, as follows: 0, no evidence of erythema or swelling; 1, erythema and mild swelling confined to the midfoot (tarsals) or ankle joint; 2, erythema and mild swelling extending from the ankle to the midfoot; 3, erythema and moderate swelling extending from the ankle to the metatarsal joint; and 4, erythema and severe swelling encompassing the ankle, foot, and digits. The severity of arthritis was determined as the sum of scores from all legs assessed by two independent observers blinded to the experimental groups.

### Histological Analysis

Mouse joint tissues were fixed in 4% paraformaldehyde (Sigma-Aldrich, St. Louis, MO, USA), decalcified in a histological decalcifying solution (Calci-Clear Rapid; National Diagnostics, Atlanta, GA, USA), and embedded in paraffin wax for histological analyses. Sections (7 μm) were prepared and stained with hematoxylin (YD Diagnostics, Yongin, Korea), eosin (Muto Pure Chemicals Co., Ltd., Tokyo, Japan), and safranin O (Sigma-Aldrich). Cartilage damage was marked as described previously (25).

### Immunohistopathological Analysis of Arthritis

Joint tissues were incubated overnight at 4°C with primary antibodies against TNFα (Abcam, Cambridge, UK), IL-17 (Abcam), IL-6 (Abcam), and IL-1β (Novus Biologicals, Littleton, CO, USA). Subsequently, samples were incubated with a biotinylated streptavidin–peroxidase complex for 1 h, and the signals were developed using chromogen 3,3′-diaminobenzidine (Thermo Scientific, Rockford, IL, USA). The sections were examined under a photomicroscope (Olympus, Tokyo, Japan). The number of positive cells in high-power digital images (magnification, ×400) was counted using Adobe Photoshop software (Adobe, San Jose, CA, USA). Stained cells were counted independently by three observers, and the mean values were evaluated.

### Measurement of Anti-CII Antibodies and Immunoglobulin

Serum was gathered 7 weeks after the first immunization for concentration of the IgA, IgM, IgG2a, and collagen-specific IgG2a levels, and the serum was stored at −70°C until further use. The levels of the IgA, IgM, IgG2a, and collagen-specific IgG2a antibodies in the serum were measured using ELISA (Bethyl Laboratories, Montgomery, TX, USA).

### Flow Cytometry Analysis

Cytokine expression in mice was analyzed *via* intracellular staining with the following antibodies: fluorescein isothiocyanate-conjugated anti-IL-17, APC-conjugated anti- interferon-gamma (IFN-γ), and PerCP-conjugated anti-CD4. Before staining, cells were stimulated for 4 h with phorbol myristate and ionomycin in the presence of GolgiStop (BD Biosciences). To analyze regulatory T (Treg) populations, splenocytes were stained with PerCP-conjugated anti-CD4, APC-conjugated anti-CD25, and PE-conjugated anti-Foxp3 antibodies. T helper and Treg populations were determined using specific antibodies (eBioscience, San Diego, CA, USA). After surface staining for 30 min, the cells were permeabilized with Cytofix/Cytoperm solution (BD Biosciences). Thereafter, the cells were intracellularly stained with fluorescent antibodies. Flow cytometry was performed with the aid of a cytoFLEX Flow Cytometer (Beckman Coulter, Brea, CA, USA), and flow cytometry data were analyzed using FlowJo (Tree Star, Ashland, OR, USA).

### Isolation and Stimulation of Peripheral Blood Mononuclear Cells

PBMCs were isolated from healthy donors. Briefly, blood from donors was mixed with PBS, and the mixture was cautiously layered onto 10 ml of Ficoll Plaque Plus solution (GE Healthcare Life Sciences, Marlborough, MA, USA). Blood samples were centrifuged for 30 min at 2,500 rpm and 20°C to separate blood contents. After centrifugation, the layer containing PBMCs was collected, and the cells were washed with PBS and cultured in RPMI medium containing 10% fetal bovine serum. PBMCs were incubated with plate-bound anti-CD3 (0.5 μg/ml) and then subjected to IL-17 analysis by ELISA for 3 days or subjected to RNA sequencing and real-time polymerase chain reaction analysis for 2 days.

### Enzyme-Linked Immunosorbent Assay

The supernatant was collected 3 days after *B. longum* RAPO or vehicle treatment with anti-CD3 (0.5 μg/ml). IL-17 was analyzed by sandwich enzyme-linked immunosorbent assay (ELISA; IL-17 DuoSet ELISA; R&D Systems, Lille, France). The absorbance at 450 nm was determined using an ELISA microplate reader (Molecular Devices).

### mRNA Sequencing Data

We preprocessed the raw reads from the sequencer to remove low-quality and adapter sequence before analysis and aligned the processed reads to *Homo sapiens* (*hg38*) using HISAT v2.1.0 (26). HISAT utilizes two types of indexes for alignment (a global, whole-genome index and tens of thousands of small local indexes). These two types of indexes are constructed using the same BWT (Burrows–Wheeler transform) and a graph FM index (GFM) as Bowtie2. Because of its use of these efficient data structures and algorithms, HISAT generates spliced alignments several times faster than Bowtie and BWT that are widely used. The reference genome sequence of *Homo sapiens* (*hg38*) and the annotation data were downloaded from the UCSC table browser (http://genome.uscs.edu). Transcript assembly and abundance estimation used StringTie (27, 28). After alignment, StringTie v1.3.4d was used to assemble aligned reads into transcripts and to estimate their abundance. It provides the relative abundance estimates as read count values of transcript and gene expressed in each sample. Also, transcript assembly of known transcripts, novel transcripts, and alternative splicing transcripts was processed by StringTie v2.1.3b.

### Statistical Analysis of Gene Expression Level

The relative abundances of gene were measured in read count using StringTie. We performed statistical analysis to find differentially expressed genes using the estimates of abundances for each gene in the samples. Genes with one more than zeroed read count values in the samples were excluded. To facilitate log2 transformation, 1 was added to each read count value of filtered genes. Filtered data were log2-transformed and subjected to TMM normalization. Statistical significance of the differential expression data was determined using exactTest using edgeR and fold change in which the null hypothesis was that no difference exists among groups. False discovery rate (FDR) was controlled by adjusting *p*-value using the Benjamini–Hochberg algorithm. For the DEG set, hierarchical clustering analysis was performed using complete linkage and Euclidean distance as a measure of similarity. Gene enrichment and functional annotation analysis and pathway analysis for significant gene list were performed based on gProfiler (https://biit.cs.ut.ee/gprofiler/gost) and KEGG pathway (http://www.genome.jp/kegg/pathway.html).

### Real-Time Polymerase Chain Reaction

Total RNA was extracted using TRI reagent (Molecular Research Center, USA) and cDNA was synthesized with the Dyne First Strand cDNA Synthesis Kit (Dyne Bio, South Korea) according to the protocol of the manufacturer. mRNA was quantified using the StepOnePlus™ Real-Time PCR systems (Applied Biosystems, USA) with SensiFAST SYBR Hi-ROX (Bioline, USA). mRNA levels were normalized to that of β-actin. The following primers were used: β-actin 5′-GGACTT CGAGCAAGAGATGG-3′ and 5′-TGTGTTGGGGTACAGG TCTTTG-3′; IL-17A 5′-CAACCGATCCACCTCACC TT-3′ and 5′-GGCACTTTGCCTCCCAGAT-3′; RORC 5′-AGTCGGAAGGCAAGATCAGA-3′ and 5′-CAAGAGAGGTTCTGG GCAAG-3′; IL-21 5′-TGTGAATGACTTGGACCCTGAA-3′ and 5′-AAACAG GAAATAGCTGACCACTCA-3′; IRF-4 5′-CCTGCAAGCTCTTTGACACA-3′ and 5′-GAGTCACCTGGAATCTTGGC-3′; and IL-23R 5′-AGGTACTGGCAGCCTTGGAGTT-3′ and 5′-CCCTGTAGAGATGGAAGCAACTG-3′.

### Statistical Analysis

Non-parametric statistical tests (Mann–Whitney test and Kruskal–Wallis test) were used to compare the alpha diversity of each group and the relative abundance of each taxon. Pairwise permutational analysis of variance (PERMANOVA) was used to compare beta diversity between groups. Spearman correlation analysis was used to analyze the relationship between the relative abundance of each taxon and RF levels. Data were analyzed using GraphPad Prism (V.5 for Windows; GraphPad Software Inc., La Jolla, CA, USA) and are presented as means ± standard deviations. Comparisons of numerical data between two groups were performed using Student’s *t*-test or the Mann–Whitney *U*-test. Differences in the mean values of more than two groups were assessed using analysis of variance with a *post-hoc* test. *p*-values <0.05 (two-tailed) were considered to indicate statistical significance.

## Results

### Comparison of Bacterial Alpha and Beta Diversities

To examine the relationship between RF levels and changes in intestinal microbial diversity, we performed gut microbiome profiling through 16S rRNA sequencing of fecal samples from 77 RF-positive patients, 16 RF-negative patients, and 16 healthy volunteers (**Table 1**). Raw sequences were filtered and merged, and 1,284,582 reads were grouped into operational taxonomic units (OTUs) based on 97% similarity, with a mean of 11,059 across 110 samples. Alpha diversity (Faith’s PD), OTU number, Pielou’s evenness, and Shannon’s diversity index did not differ significantly between the RF-negative, RF-low, and RF-high groups (**Supplementary Figures 1A–D**). However, there were significant differences in beta diversity between RF-negative and RF-low RA patients and healthy subjects (**Supplementary Figures 1F, G**). Notably, RF-negative RA patients had a significantly lower Shannon index compared with healthy subjects (**Supplementary Figure 1A**). RF-low individuals exhibited a significantly lower Shannon index and OTU number compared with healthy volunteers (**Supplementary Figures 1A, B**). Jaccard distance-based beta diversity analysis revealed significant differences in the microbial community between healthy subjects and RF-low RA patients (*p* = 0.027, PERMANOVA; **Supplementary Figure 1F**), as well as between healthy subjects and RF-high RA patients (*p* = 0.010, PERMANOVA; **Supplementary Figure 1F**). Unweighted UniFrac distance-based microbial structure analysis showed significant differences between healthy subjects and RF-negative RA patients (*p* = 0.045, PERMANOVA; **Supplementary Figure 1G**), as well as between healthy subjects and RF-high RA patients (*p* = 0.003, PERMANOVA; **Supplementary Figure 1G**). Other beta diversity matrices did not differ significantly among the groups (**Supplementary Figures 1E, H**).

### Association Between RF Levels and Microbial Composition

**Figure 1:**
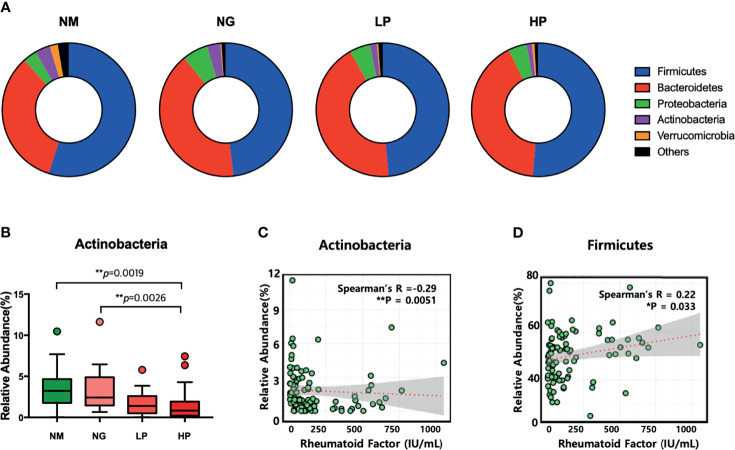
Microbial composition at the phylum level in patients with different rheumatoid factor (RF) levels. **(A)** Comparison of intestinal microflora composition at the phylum level. **(B)** Relative abundance of Actinobacteria. Comparisons in relative abundance were performed using the Kruskal–Wallis test. **(C)** Correlation between the relative abundance of Actinobacteria and RF levels. **(D)** Correlation between the relative abundance of Firmicutes and RF levels. *p < 0.05; **p < 0.01. NM, normal control; NG, rheumatoid arthritis (RA) patients showing 20 or less of RF level; LP, RA patients showing over 20 and not exceeding 60 of RF level; HP, RA patients showing over 60 of RF level.

**Figure 2:**
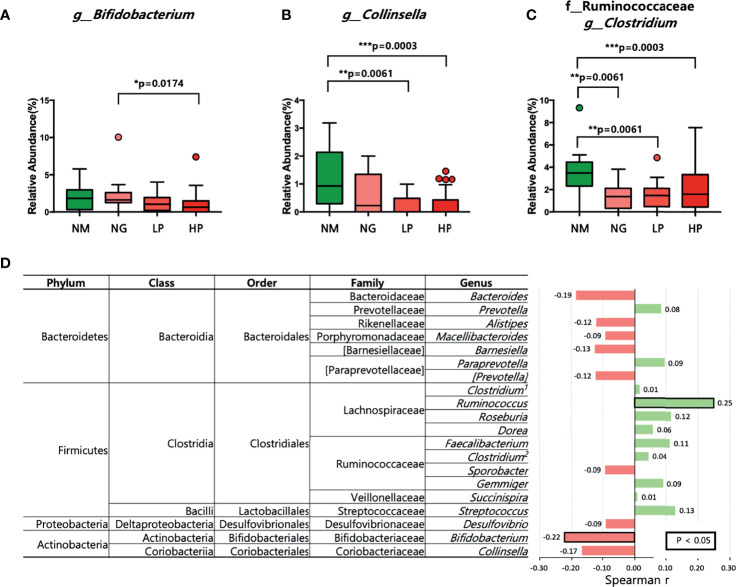
Microbial composition at the genus level in patients with different RF levels. **(A–C)** Comparison of the relative abundance of **(A)** *Bifidobacterium*, **(B)** *Collinsella*, and **(C)** *Clostridium* using the Kruskal–Wallis test. **(D)** Correlations between the relative abundance of each of the 20 most abundant genera and RF levels. Positive correlations are shown in green, and negative correlations are shown in pink. The numbers in bold indicate statistically significant correlations (*p* < 0.05). *p < 0.05; **p < 0.01; ***p < 0.001. NM, normal control; NG, RA patients showing 20 or less of RF level; LP, RA patients showing over 20 and not exceeding 60 of RF level; HP, RA patients showing over 60 of RF level.

Although there were no differences in microbial diversity in RA patients according to RF level, the Kruskal–Wallis test revealed significant differences in the composition of individual taxa. We identified 11 differentially abundant taxa at the false discovery rate of 10%; 10 taxa were enriched in healthy volunteers, and one taxon was enriched in RA patients (**Table 2**). At the phylum level, Actinobacteria were less abundant in RF-high RA patients than in healthy individuals and RF-negative RA patients (**Figures 1A, B**). The relative abundance of Actinobacteria in all RA patients was negatively correlated with RF level (*R* = −0.29, *p* = 00051; **Figure 1C**). By contrast, the abundance of the phylum Firmicutes in all RA patients was positively correlated with RF levels (*R* = 0.22, *p* = 0.0322; **Figure 1D**). Within the Actinobacteria phylum, *Bifidobacterium* were significantly more abundant in RF-negative than in RF-high RA patients (**Figure 2A**). Another genus of the Actinobacteria phylum, *Collinsella*, was more abundant in healthy volunteers than in RF-negative or RF-high RA patients; however, *Collinsella* abundance did not differ significantly among RA patients (**Figure 2B**). Similarly, *Clostridium* species were more abundant in healthy individuals than in the different RA patient groups, although their abundance was similar across RA patient groups (**Figure 2C**). Compared with RF-negative RA patients and healthy individuals, RF-high RA patients exhibited a lower prevalence of the genera *Bifidobacterium*, *Collinsella*, and *Clostridium* (**Supplementary Figures 3A–C**). The prevalence of *Veillonellaceae* family was the highest among the control and RA patients (**Supplementary Figure 3D**).

**Table 2:** Differentially enriched taxa in rheumatoid patients and healthy subjects.

Taxa		*p*-value	FDR	NM mean (%)	NG mean (%)	LP mean (%)	HP mean (%)	NM prevalence (%)	NG prevalence (%)	LP prevalence (%)	HP prevalence (%)
Enriched taxa in NM											
Phylum	Actinobacteria	0.0001	0.0006	3.69^a^	3.43^a^	1.7^ab^	1.34^b^	87.50	100.00	83.33	81.13
Class	Actinobacteria	0.0051	0.0281	2.04^ac^	2.27^ab^	1.26^ac^	0.99^c^	87.50	93.75	79.17	71.70
	Coriobacteriia	0.0000	0.0004	1.65^a^	1.16^ab^	0.44^bc^	0.35^cd^	87.50	75.00	50.00	47.17
Order	Bifidobacteriales	0.0084	0.0546	1.97^ab^	2.24^b^	1.21^ab^	0.99^ac^	87.50	93.75	79.17	71.70
	Coriobacteriales	0.0000	0.0005	1.65^a^	1.16^ab^	0.44^bc^	0.35^cd^	87.50	75.00	50.00	47.17
Family	Bifidobacteriaceae	0.0084	0.0756	1.97^ab^	2.24^b^	1.21^ab^	0.99^ac^	87.50	93.75	79.17	71.70
	Coriobacteriaceae	0.0000	0.0007	1.65^a^	1.16^ab^	0.44^bc^	0.35^cd^	87.50	75.00	50.00	47.17
Genus	*Bifidobacterium*	0.0084	0.0840	1.97^ab^	2.24^b^	1.21^ab^	0.99^ac^	87.50	93.75	79.17	71.70
	*Collinsella*	0.0005	0.0094	1.14^a^	0.58^ab^	0.26^bc^	0.24^bc^	81.25	50.00	41.60	33.96
	*Clostridium*	0.0088	0.0587	3.42^a^	1.39^b^	1.52^b^	2.03^b^	93.75	93.75	83.33	83.02
Enriched taxa in RA											
Family	Veillonellaceae	0.0156	0.0936	1.34^ab^	1.73^bc^	3.14^bc^	3.03^c^	62.50	81.25	79.17	90.57

Comparisons were analyzed using Kruskal–Wallis tests. The different alphabet characters indicate statistically significant differences of each value among NM, NG, LP, and HP with p-value less than 0.05. The FDR was calculated with Benjamini–Hochberg procedure.

Among the genera of the Actinobacteria phylum, the genus *Bifidobacterium* exhibited a negative correlation with RF levels (*R* = −0.22, *p* = 0.0326; **Figure 2D**). Furthermore, the abundance of the genus *Ruminococcus* was positively correlated with RF levels (*R* = 0.25, *p* = 0.0161; **Figure 2D**).

### *Bifidobacterium longum* RAPO Therapy Reduced the Severity of CIA

**Figure 3:**
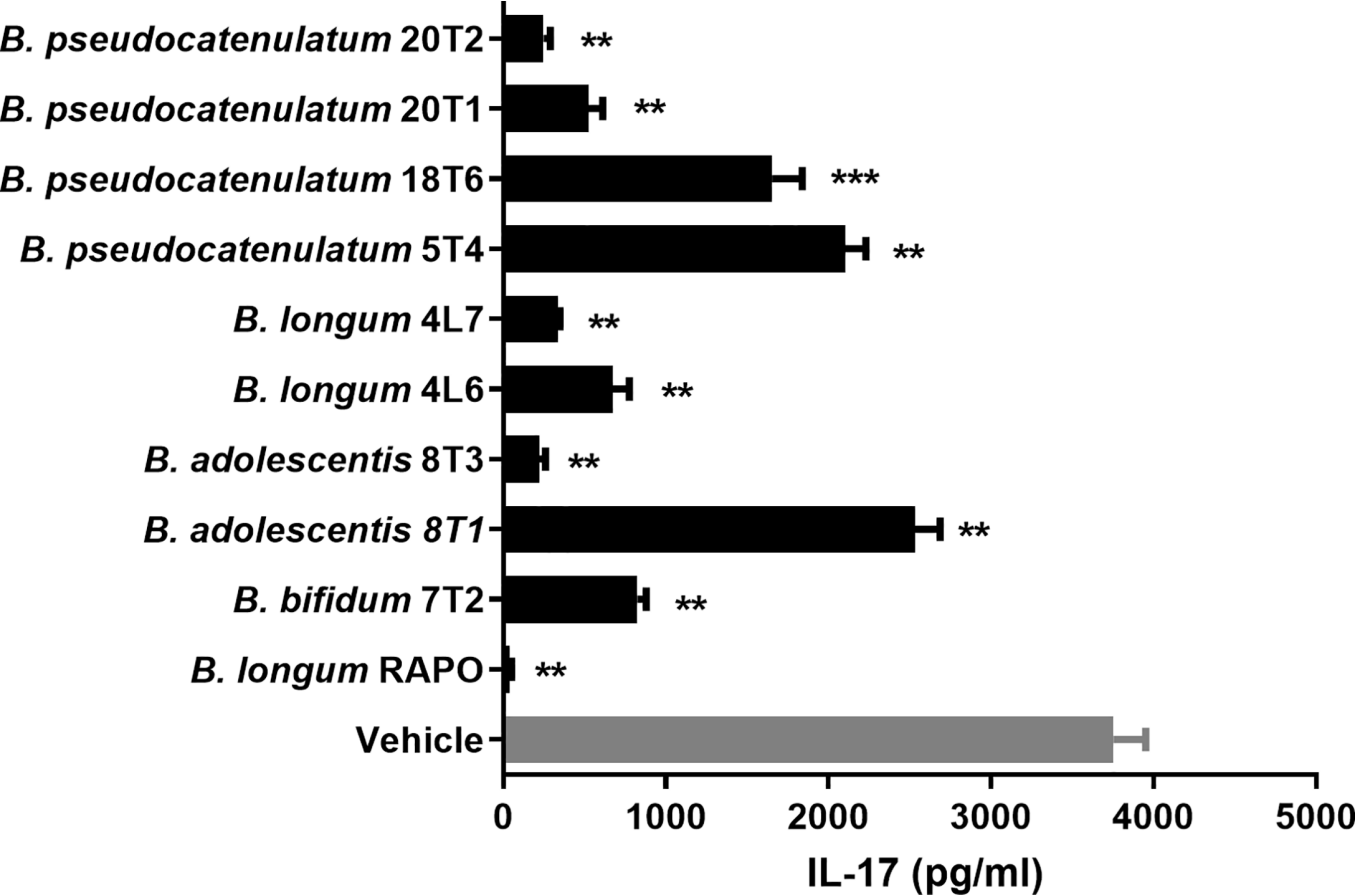
*Bifidobacterium longum* RAPO suppresses IL-17 expression in human PBMCs. PBMCs were stimulated with anti-CD3 for 72 h. IL-17 levels in the culture supernatant were analyzed using ELISA. Data are presented as the means ± standard deviations (SDs) from three independent experiments. **p < 0.03; ***p < 0.01.

*Bifidobacterium* genera can be cultivated despite being anaerobes and are widely known for immune regulation ability. Hence, we decided to focus on *Bifidobacterium* genus among the differentially abundant genera for further study. To identify the most potent *Bifidobacterium* strain among the *Bifidobacterium* isolated from healthy individuals and RA patients, we treated PBMCs (stimulated with anti-CD3) with *B. pseudocatenulatum* 20T2, *B. pseudocatenulatum* 20T1, *B. pseudocatenulatum* 18T6, *B. pseudocatenulatum* 5T4, *B. longum* 4L7, *B. longum* 4L6, *B. adolescentis* 8T3, *B. adolescentis* 8T1, *B. bifidum* 7T2, and *B. longum* RAPO. Among these strains, *B. longum* RAPO exhibited the most potent inhibitory effect on IL-17 secretion (**Figure 3**).

**Figure 4:**
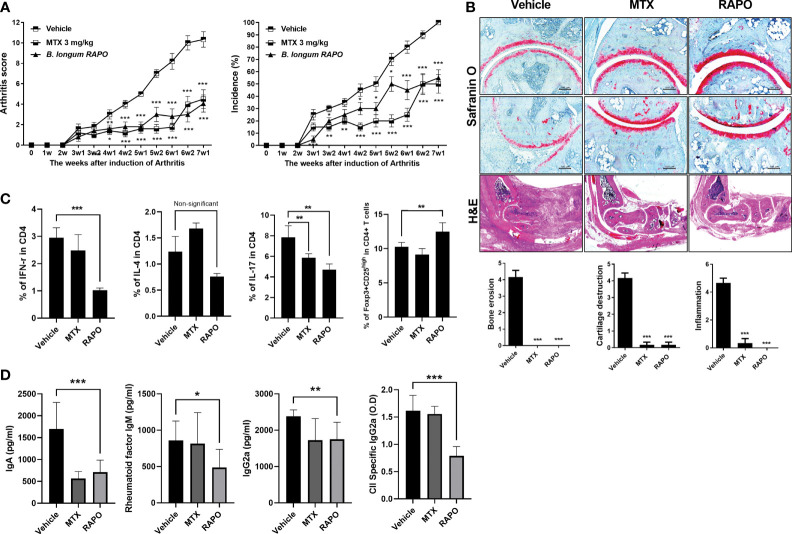
*Bifidobacterium longum* RAPO alleviates RA in collagen-induced arthritis (CIA) mice. CIA mice (*n* = 5 per group) were orally administered *B. longum* RAPO (1 × 10^8^ CFU/mouse) or methotrexate (MTX; 3 mg/kg) once daily for 7 weeks after the immunization boost. **(A)** Reductions in arthritis score and arthritis incidence in CIA mice treated with *B. longum* RAPO. Effects of *B. longum* RAPO on RA development in CIA mice. **(B)** Tissue specimens were acquired from the hind paw joints of mice and stained with hematoxylin and eosin and safranin O. Representative histological quality and histological grades are shown. **(C)** Splenocytes isolated at 7 weeks after immunization were stimulated with phorbol myristate, ionomycin, and GolgiStop for 4 h. The percentages of Th1 (CD4^+^IFN-γ^+^), Th2 (CD4^+^IL-4^+^), and Th17 (CD4^+^IL-17^+^) cells were analyzed using flow cytometry, including also Treg (CD4^+^CD25^high^ Foxp3^+^). **(D)** Levels of IgA, IgM, IgG2a, and anti-CII-specific IgG2a antibodies in the serum of CIA mice at 7 weeks after the first immunization. Data are presented as the means ± SDs. *p < 0.05; **p < 0.01; ***p < 0.001 (*vs*. vehicle-treated group).

To evaluate the effects of *B. longum* RAPO in CIA, we orally administrated 1 × 10^8^ CFU/mouse *B. longum* RAPO alone, methotrexate 3 mg/kg alone, or vehicle to CIA mice at 3 weeks after immunization. Mice developed RA at 21 days after immunization; however, oral administration of *B. longum* RAPO to CIA mice decreased CIA, as demonstrated by a significantly reduced arthritis score compared with the vehicle-treated mice (**Figure 4A**). In mice that were given oral administration of *B. longum* RAPO, the severity of arthritis damage was attenuated, and cartilage protection was improved, compared with the control mice. Inflammation score, bone damage, and cartilage damage were also significantly decreased in mice treated with *B. longum* RAPO (**Figure 4B**). Importantly, the frequencies of CD4^+^ T cells producing IFN-γ and IL-17 were significantly lower in mice treated with *B. longum* RAPO than in control mice treated with the vehicle. Furthermore, administration with *B. longum* RAPO resulted in an increased Treg population, compared with the control (**Figure 4C**). Serum IgA, rheumatoid factor IgM, IgG2a, and CII-specific IgG2a levels were reduced by the administration of *B. longum* RAPO (**Figure 4D**). *, *p* < 0.05; **, *p* < 0.01; ***, *p* < 0.001 (*vs*. vehicle-treated group).

### Effect of *Bifidobacterium longum* RAPO on Obese CIA

**Figure 5:**
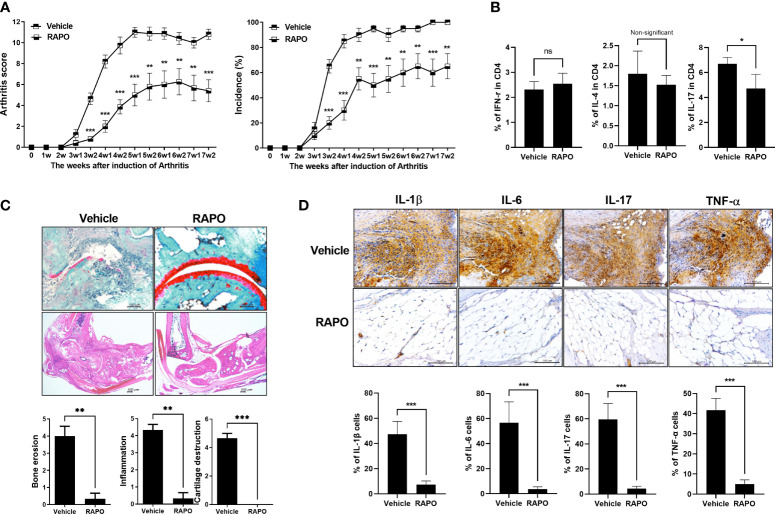
Effects of *Bifidobacterium longum* RAPO in obese CIA mice. Mice were administered orally with *B. longum* RAPO (1 × 10^8^ CFU/mouse) once daily for 7 weeks after the immunization boost. **(A)** Arthritis score and incidence of *B. longum* RAPO-treated mice compared with those of obese CIA mice (*n* = 5 for each group). **(B)** *B. longum* RAPO reduces IL-17 expression in CD4 T cells from the spleen of mice with obese CIA. Flow cytometry of Th1 cells (IFN-r^+^CD4^+^), Th2 cells (IL-4^+^CD4^+^), and Th17 cells (CD4^+^IL17^+^) from the spleen of mice with obese CIA. **(C)** Effect of *B. longum* RAPO on RA in mice with obese CIA. Tissue from the hind paw joints was stained with hematoxylin and eosin, as well as safranin O. **(D)** *B. longum* RAPO inhibits the proinflammatory cytokines IL-1β, IL-6, IL-17, and TNFα in CIA mice. Representative immunohistochemistry images showing that *B. longum* RAPO alleviates RA in obese CIA mice. Synovium sections treated with a vehicle, *B. longum* RAPO, or vehicle were stained for IL-1β, IL-6, IL-17, and TNFα. Scale bar, 100 μm. *p < 0.05; **p < 0.01; ***p < 0.001 (*vs*. vehicle-treated group).

An obese arthritis model was reported according to a previous report (29). Obese CIA mice had a more serious state of disease and a higher arthritis score compared with CIA. To investigate the effect of *B. longum* RAPO in obese CIA, obese CIA-induced RA mice were fed the HFD for 7 weeks. There was a significant decrease in the arthritis score and incidence in the *B. longum* RAPO-administered mice compared with the vehicle-treated obese CIA mice (**Figure 5A**). We then performed effector T-cell staining on splenocytes from *B. longum* RAPO- and vehicle-treated mice. Flow cytometry analysis of splenocytes showed that the Th17 (IL-17^+^ in CD4^+^ T cells) population was reduced by the administration of *B. longum* RAPO; on the other hand, the populations of Th1 (IFN-γ^+^ in CD4 T cells) and Th2 (IL-4^+^ in CD4^+^ T cells) did not differ significantly between *B. longum* RAPO- and vehicle-treated mice with obese CIA (**Figure 5B**). In mice that received *B. longum* RAPO, the severity of arthritis was attenuated, and cartilage conservation was improved, compared with the vehicle-treated mice (**Figure 5C**). We also analyzed *B. longum* RAPO effects on inflammatory cytokine staining of joints which showed that *B. longum* RAPO treatment diminished the levels of IL-1β, IL-6, IL-17, and TNFα (**Figure 5D**). *, *p* < 0.05; **, *p* < 0.01; ***, *p* < 0.001 (*vs*. vehicle-treated group).

### Inhibition of Th17 Cells by *Bifidobacterium longum* RAPO Treatment on Human PBMCs *In Vitro*

**Figure 6:**
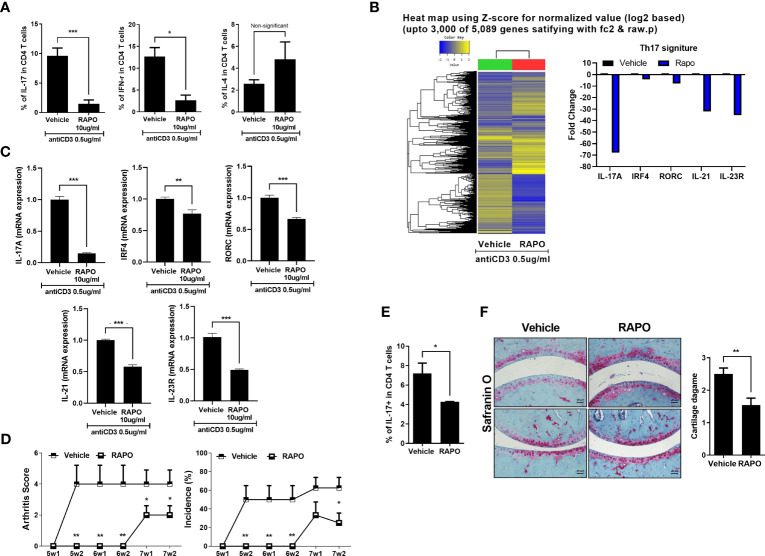
Effects of *Bifidobacterium longum* RAPO in human PBMCs and avatar mice of RA patient. **(A)** The PBMCs of the RA patient were cultured with anti-CD3 antibody for 72 h and the resulting Th1 cells (IFN-r^+^CD4^+^), Th2 cells (IL-4^+^CD4^+^), and Th17 cells (CD4^+^IL17^+^) were analyzed. **(B)** The PBMCs of the RA patient were cultured with anti-CD3 antibody for 48 h. Hierarchical cluster heatmap of the PBMC-stimulated anti-CD3 antibody of RA patient treated with *B. longum* RAPO or vehicle. The expression of Th17 pathway was analyzed by RNA sequencing. Significantly differentially expressed gene and significant differences in Th17 pathway activities. The fold change of Th17-related genes decreased in the treatment of the *B. longum* RAPO. **(C)** Relative mRNA expression of Th17 pathway genes was analyzed by real-time PCR. **(D)** NSG mice were administered with *B. longum* RAPO (1 × 10^8^ CFU/mouse) once daily for 7 weeks after the sensitization injection. **(E)** Splenocytes from the avatar mice of RA patient treated with *B. longum* RAPO. The cells were stained with Abs against CD4, IL-17. A graph from a representative experiment showing the frequency of IL-17^+^ cells in CD4 T cells. **(F)** Joint sections from the avatar mice of RA patient with *B. longum* RAPO-treated mice were stained with safranin O. *p < 0.05; **p < 0.01; ***p < 0.001 (*vs*. vehicle-treated group).

The PBMCs of RA patients were cultured under anti-CD3 0.5 μg/ml conditions with *B. longum* RAPO for 72 h. Flow cytometry showed that *B. longum* RAPO treatment significantly suppressed Th1 and Th17 cell proliferation (**Figure 6A**). We next analyzed by RNA sequencing the gene expression profiles of RA PBMC treatment with *B. longum* RAPO in the presence of anti-CD3 0.5 μg/ml for 48 h. When demonstrated by absent/present classification and using at least two-fold difference in expression as the cutoff, 5,089 genes were differentially expressed in vehicle-treated RA PBMCs versus *B. longum* RAPO-treated RA PBMCs. Among those in the Th17 pathway, *Il17*, *IRF4*, *RORC*, *SIl21*, and *Il23* were downregulated by *B. longum* RAPO (**Figure 6B**). Also, Th17-related genes confirmed that *Il17*, *IRF4*, *RORC*, *SIl21*, and *Il23* were downregulated by qPCR (**Figure 6C**). *, *p* < 0.05; **, *p* < 0.01; ***, *p* < 0.001 (*vs*. vehicle-treated group).

### *Bifidobacterium longum* RAPO Ameliorates Human Avatar Arthritis Mice

Arthritis score and incidence were examined to investigate whether oral administration of *B. longum* RAPO can have an effect on human avatar arthritis mice. The engraftment of the patient PBMC was confirmed by analyzing the expression of CD4 and CD8 T cells using human antibodies (**Supplementary Figure 4D**). The *B. longum* RAPO-treated group had a lower arthritis score and incidence than the vehicle group (**Figure 6D**).

Also, it was confirmed that Th17 cells were effectively inhibited like CIA and obese CIA (**Figure 6E**). Histological analysis was performed using safranin O staining to confirm the degree of cartilage destruction in avatar mice of RA patient. The cartilage damage score was low in the *B. longum* RAPO-administered group (**Figure 6F**). **, *p* < 0.01; ***, *p* < 0.001 (*vs*. vehicle-treated group).

## Discussion

In this study, we investigated the gut microbiota composition in RA patients with varying RF levels and the effects of *B. longum* RAPO on RA. Previous studies have shown that targeted therapies can modulate the gut microbiota composition and increase the abundance of beneficial microorganisms (30). Here, we found significant differences in the gut microbiota composition between healthy individuals and RA patients with varying RF levels. In accordance with our previous findings (24), we found that RA patients had altered levels of *Collinsella* among other Actinobacteria. Species of the genus *Collinsella* have been demonstrated to modify host levels of bile acids and plasma cholesterol by producing various beneficial short-chain fatty acids, such as butyric acid, acetic acid, formic acid, and lactic acid (31–34). Chen et al. reported that the relative abundance of *Collinsella aerofaciens* is higher in RA patients (35). However, as the genus *Collinsella* consists of nine diverse species, species-specific or strain-specific effects are possible (34). Members of Actinobacteria, and the genus *Collinsella* in particular, seem to be enriched in the gut microbiota of RA patients.

Several studies have profiled the gut microbiome in RA patients, mainly in early-stage RA patients. Scher et al. found that *Prevotella copri* was more abundant in NORA patients than in healthy subjects, and Alpizar-Rodriquez et al. confirmed that *P. copri* was enriched in individuals at risk of RA (19, 36). However, microbiome profiling studies of RA patients with different levels of clinical indices remain limited. The presence of RF is associated with RA severity independent of anti-citrullinated protein antibodies (16). To elucidate the relationship between RF and gut microbiome composition, we controlled for anti-citrullinated protein antibodies and other clinical indices other than RF. The gut microbiome of RA patients with different RF levels exhibited significant differences in terms of alpha and beta diversities. Microbial composition differed profoundly among the groups. Notably, Actinobacteria and *Bifidobacterium* were less abundant in RF-positive RA patients than in their RF-negative counterparts, with RA patients with RF >60 IU/ml exhibiting the lowest relative abundance of those taxa. Moreover, the Actinobacteria phylum and *Bifidobacterium* genus exhibited a strong negative correlation with RF levels, suggesting that these taxa may be involved in RA in an RF-dependent manner. Hence, in RA patients with high RF titers, a low abundance of *Bifidobacterium* could be considered a risk factor of RA.

A reduced relative abundance of *Bifidobacterium* has been reported in patients with RA and other immune-related diseases. Vogt et al. reported that bifidobacteria were less abundant in patients with Alzheimer’s disease than in healthy subjects and that the relative abundance of bifidobacteria was negatively correlated with the extent of amyloid deposition (37). Interestingly, Matson et al. showed that melanoma patients had a lower abundance of *Bifidobacterium* than did control subjects (38). Additionally, alterations in the abundance of bifidobacteria are prominent in elderly people (39–41). A large-scale association study with 18,340 individuals from 24 population-based cohorts of European, Hispanic, Middle Eastern, Asian, and African ancestries also identified that the association between the lactase (LCT) gene locus and the abundance of *Bifidobacterium* was age-dependent (21). These findings suggest that a reduction in the abundance of *Bifidobacterium* in patients with high RF levels may reflect an unhealthy status.

To assess whether the reduction in the abundance of *Bifidobacterium* is the cause or a consequence of RA, we administered bifidobacteria to mice with RA. *Bifidobacterium longum* RAPO was identified as the *Bifidobacterium* strain with the strongest ability to inhibit IL-17 production. In mice, *B. longum* RAPO treatment significantly reduced RA incidence, RA score, and the production of autoantibodies. Importantly, administration of *B. longum* RAPO in RA mice significantly inhibited joint inflammation and prevented bone damage, cartilage damage, and loss of large intestine tissue. These findings strongly suggest that oral administration of *B. longum* RAPO may alleviate RA and other autoimmune diseases.

Additionally, oral administration of *B. longum* RAPO significantly reduced the frequencies of IFN-γ-positive and IL-17-positive CD4^+^ T cells, suggesting that *B. longum* RAPO components may modulate the development and function of Th17 cells and regulatory T cells.

Through RNA sequencing analysis, we also indicated that *B. longum* RAPO treatment inhibited the expression of *IL-17A*, *IRF4*, *RORC*, and *IL-23R*. These results significantly reduced IL-17 in collagen-induced arthritis, obese arthritis, and avatar arthritis mice. In addition, RNA sequencing results confirmed that it regulates the increase and decrease of genes related to necroptosis, autophagy, and AMPK/mTOR. Indeed, inhibition of necroptosis factor is also implicated in the treatment mechanism of rheumatoid arthritis (42, 43). Further molecular mechanism studies are needed to understand these mechanisms.

The potential therapeutic effects of probiotic bacteria in RA have been previously reported. Nevertheless, this is the first study to report the anti-RA effects of a bacterial strain identified through high-throughput screening. Additionally, there are many discrepancies in the findings of previous studies of the therapeutic effects of probiotic bacteria in RA. For example, Pan et al. reported that *Lactobacillus casei* (ATCC334) restored gut microbial dysbiosis and prevented bone destruction in a rodent model of RA (44). Additionally, *Lactobacillus helveticus* SBT2171 has been reported to alleviate RA in a CIA mouse model (45). In patients with RA, supplementation of a probiotic formulation containing *Lactobacillus acidophilus*, *L. casei*, and *B. bifidum* significantly improved the DAS28 (46). By contrast, Pineda et al. reported that oral administration of *Lactobacillus rhamnosus* GR-1 and *Lactobacillus reuteri* RC-14 in RA patients did not improve the clinical features of RA (47). A meta-analysis showed that although probiotics could lower the levels of the proinflammatory cytokine IL-6 in RA patients, they failed to alleviate the clinical manifestations of RA (48). Even though these discrepancies among studies may have resulted from differences in the strain or dose used, the lack of mechanistic evidence from well-designed studies also hinders the establishment of effective probiotic regimens. Additionally, most previous studies have focused on strains known to inhibit inflammation. By contrast, we conducted unbiased screening based on microbiome profiling data and identified a novel *Bifidobacterium* strain that alleviates RA.

An important limitation of this study is the lack of evidence in humans. Although *B. longum* RAPO exerted strong anti-RA effects in mice, these effects should be confirmed in human studies. Therefore, well-designed, controlled clinical trials are required to investigate the safety and efficacy of *B. longum* RAPO in patients with RA or other immune-related diseases involving RF, such as Sjögren’s syndrome and systemic lupus erythematosus. In conclusion, we identified *B. longum* RAPO as a potential anti-RA strain through gut microbiome profiling of RA patients and investigated its RA-modulating effects in a mouse RA model. Our findings strongly suggest that *B. longum* RAPO supplementation may alleviate RA by inhibiting the production of proinflammatory mediators. Future studies are required to confirm these findings in different mouse models, as well as investigate the safety and efficacy of *B. longum* RAPO in patients with RA and other autoimmune disorders.

## Data Availability Statement

The 16sRNA sequence data presented in the study are deposited in the Sequence Read Archive (SRA) repository, accession number PRJNA759926. The RNA-sequencing data presented in the study is deposited in the GEO repository, accession number GSE183154.

## Ethics Statement

The studies involving human participants were reviewed and approved by the Institutional Review Board of Seoul St. Mary’s Hospital, The Catholic University of Korea (approval ID: KC17TNSI0570). The patients/participants provided their written informed consent to participate in this study. All experimental procedures were evaluated and conducted in accordance with the protocols approved by the Animal Research Ethics Committee at the Catholic University of Korea (Permit Number: CUMC 2019-0242-01). All procedures performed in this study followed the ethical guidelines for animal use.

## Author Contributions

All authors were involved in drafting the article or revising it critically for important content. M-LC, GJ, and S-HP had full access to all the data in the study and took responsibility for the integrity of the data and the accuracy of the data analysis. Conception and design of the study: YJ and JJ. Acquisition of data: YJ, JJ, S-YL, HN, JC, K-HC, SL, AL, and S-JP. Analysis and interpretation of data: J-WK, MP, and BK. Drafting the article: YJ and JJ. Revising the article critically: M-LC, GJ, and S-HP. All authors contributed to the article and approved the submitted version.

## Funding

This research was supported by the Bio & Medical Technology Development Program of the National Research Foundation (NRF) and funded by the Korean Government (MSIT) (No. NRF-2017M3A9F3041045).

## Conflict of Interest

Authors YJ, S-JP, MP, BK and GJ was employed by company BIFIDO Co., Ltd.

The remaining authors declare that the research was conducted in the absence of any commercial or financial relationships that could be construed as a potential conflict of interest.

## Publisher’s Note

All claims expressed in this article are solely those of the authors and do not necessarily represent those of their affiliated organizations, or those of the publisher, the editors and the reviewers. Any product that may be evaluated in this article, or claim that may be made by its manufacturer, is not guaranteed or endorsed by the publisher.

## Supplementary Material

Supplementary Figure 1 Comparison of alpha and beta diversity among the healthy control people and RA patients grouped according to the RF level. Comparison of **(A)** Shannon index diversity, **(B)** number of observed OTUs, **(C)** Faith’s PD and **(D)** pielou’s evenness were performed for NM, NG, LP, and HP groups using Kruskal-Wallis tests. Lines inside the box represent the median, while whiskers represent the lowest and highest values within the 1.5 interquartile range (IQR). Outliers and individual sample values are shown as dots. Principal coordinate plot based on the **(E)** Bray Curtis, **(F)** Jaccard, **(G)** Unweighted-UniFrac distance matrix, and (H) Weighted UniFrac.

Supplementary Figure 2 Microbial composition at the top 20 expressed genera in patients with different RF levels. **(A–G)** Comparison of the relative abundance of genera under Bacteroidetes phylum, **(H–P)** Comparison of the relative abundance of genera under Firmicutes phylum, **(Q)** Comparison of the relative abundance of genera under Proteobacteria phylum. The numbers in bold indicate statistically significant correlations (*p* < 0.05). *, *p* < 0.05; **, *p* < 0.01; ***, *p* < 0.001. NM: Normal control, NG: RA patients showing 20 or less of RF level, LP: RA patients showing over 20 and not exceeding 60 of RF level, HP: RA patients showing over 60 of RF level.

Supplementary Figure 3 Microbial composition at the genus level in patients with different RF levels. Prevalence of **(A)** *Bifidobacterium*, **(B)** *Collinsella*, **(C)** *Clostridia*, and **(D)** *Veillonellaceae.* Prevalence was expressed as the percentage of samples containing each bacterial genus. The English in this document has been checked by at least two professional editors, both native speakers of English. For a certificate, please see: http://www.textcheck.com/certificate/JlxAE8.
