# Supplementary material for: Therapeutic Potential of a Novel Bifidobacterium Identified Through Microbiome Profiling of RA Patients With Different RF Levels
Source: Front Immunol. 2021 Nov 15;12:736196. doi: 10.3389/fimmu.2021.736196 (PMC8634832; doi:10.3389/fimmu.2021.736196)
Supplement: Supplementary Figure 1 — Comparison of alpha and beta diversity among the healthy control people and RA patients grouped according to the RF level. Comparison of (A) Shannon index diversity, (B) number of observed OTUs, (C) Faith’s PD and (D) pielou’s evenness were performed for NM, NG, LP, and HP groups using Kruskal-Wallis tests. Lines inside the box represent the median, while whiskers represent the lowest and highest values within the 1.5 interquartile range (IQR). Outliers and individual sample values are shown as dots. Principal coordinate plot based on the (E) Bray Curtis, (F) Jaccard, (G) Unweighted-UniFrac distance matrix, and (H) Weighted UniFrac. [file DataSheet_1.docx]

**SUPPLEMENTAL MATERIALS AND METHODS**

**Bacterial preparation**

Bacterial samples (50 $\mu L$) were inoculated into 5 L of cMRS liquid medium and incubated for 20 hours at 37℃. After incubation, the bacteria were collected via centrifugation (2236HR high-speed centrifuge; Gyrozen, Gimpo, Korea) at 20℃ and 7,000 rpm. After two washes with phosphate-buffered saline (PBS), the bacterial samples were dried at 36℃ and centrifuged at 2,000 rpm for 24 hours using a Scanvac Speed Vacuum Concentrator (LaboGene Aps, Lillerød, Denmark). The dried bacterial cells were heat-inactivated at 80℃ for 30 min and then mixed with PBS. All bacteria used in this study were isolated from healthy subjects and RA patients. Donor characteristics are summarized in Supplementary Table 1.

Supplemental Table 1. Clinical features of various *Bifidobacteria* donors

| **Bacteria** | **Sex** | **Age** | **Treated drug** | **DAS28 (score)** | **CRP (mg/dL)** | **ESR (mm/hr)** | **RF** | **ACCP** |
| --- | --- | --- | --- | --- | --- | --- | --- | --- |
| *B. pseudocatenulatum* 20T3 | F | 55 | - | - | - | - | - | - |
| *B. pseudocatenulatum* 20T1 | F | 55 | - | - | - | - | - | - |
| *B. pseudocatenulatum* 18T6 | M | 24 | - | - | - | - | - | - |
| *B. pseudocatenulatum* 5T4 | F | 36 | Methotrexate, Sulfasalazine, Tocilizumab | 3.03 | 0.01 | 7 | LP | HP |
| *B. longum* 4L6 | F | 66 | Methotrexate, Tocilizumab | 0.91 | 0.02 | 2 | HP | HP |
| *B. longum* 4L8 | F | 66 | Methotrexate, Tocilizumab | 0.91 | 0.02 | 2 | HP | HP |
| *B. adolescentis* 8T1 | M | 71 | Methotrexate, Leflunomide | 2.1 | 0.07 | 11 | LP | HP |
| *B. adolescentis* 8T3 | M | 71 | Methotrexate, Leflunomide | 2.1 | 0.07 | 11 | LP | HP |
| *B. bifidum* 7T2 | F | 39 | Adalimumab | 2.03 | 0.03 | 10 | LP | HP |
| *B. longum* RAPO | F | 33 | Methotrexate | 0.98 | 0.07 | 2 | LP | HP |

Those who do not have the information about treated drug and disease parameters in this table are healthy subjects. DAS28: Disease Activity Score-28, CRP: C-reactive protein, ESR: Erythrocyte sedimentation rate, RF: Rheumatoid factor, ACCP: anti-cyclic citrullinated peptide antibody

**Supplemental Figure 1.** Comparison of alpha and beta diversity among the healthy control people and RA patients grouped according to the RF level. Comparison of (A) Shannon index diversity, (B) number of observed OTUs, (C) Faith’s PD and (D) pielou’s evenness were performed for NM, NG, LP, and HP groups using Kruskal-Wallis tests. Lines inside the box represent the median, while whiskers represent the lowest and highest values within the 1.5 interquartile range (IQR). Outliers and individual sample values are shown as dots. Principal coordinate plot based on the (E) Bray Curtis, (F) Jaccard, (G) Unweighted-UniFrac distance matrix, and (H) Weighted UniFrac.


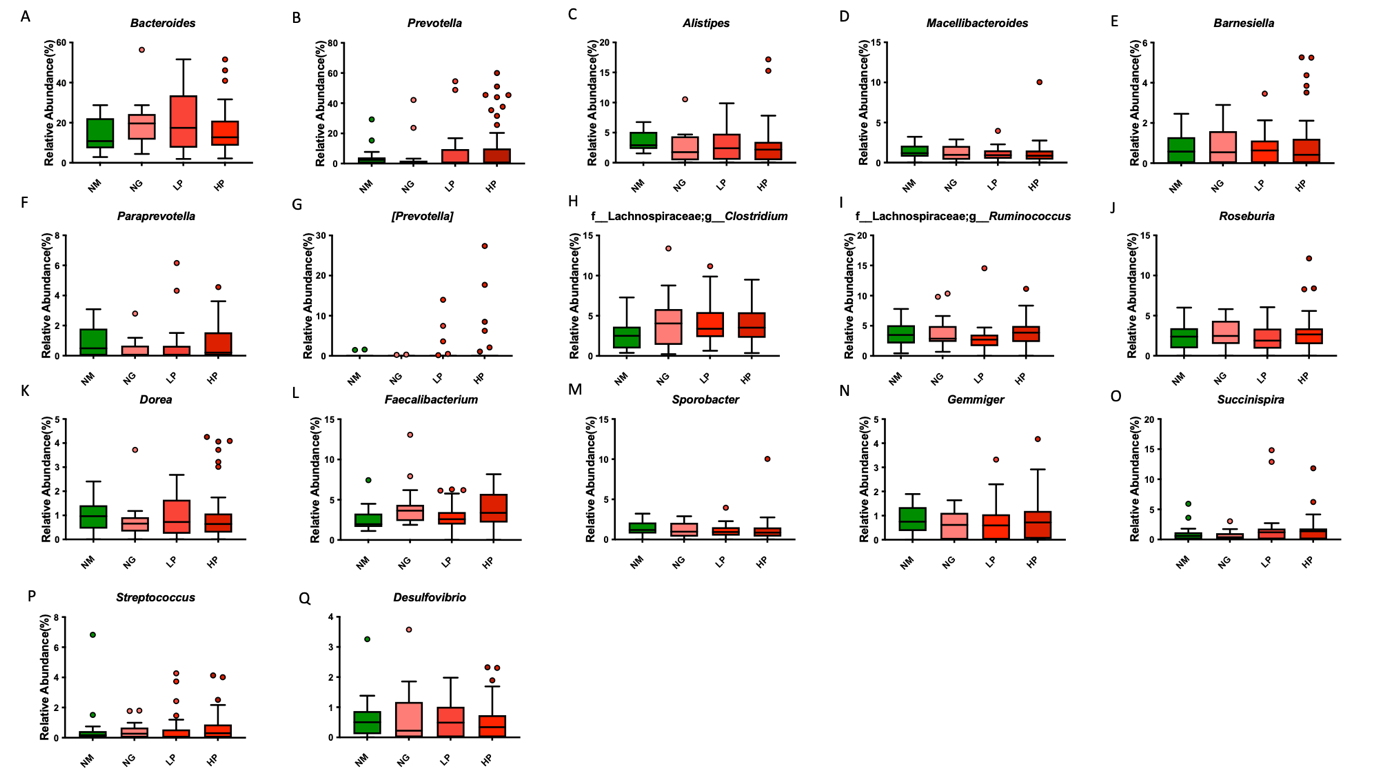


**Supplementary Figure 2:** Microbial composition at the top 20 expressed genera in patients with different RF levels. **(A–G)** Comparison of the relative abundance of genera under Bacteroidetes phylum, **(H–P)** Comparison of the relative abundance of genera under Firmicutes phylum, **(Q)** Comparison of the relative abundance of genera under Proteobacteria phylum. The numbers in bold indicate statistically significant correlations (*p* < 0.05). *, *p* < 0.05; **, *p* < 0.01; ***, *p* < 0.001. NM: Normal control, NG: RA patients showing 20 or less of RF level, LP: RA patients showing over 20 and not exceeding 60 of RF level, HP: RA patients showing over 60 of RF level.


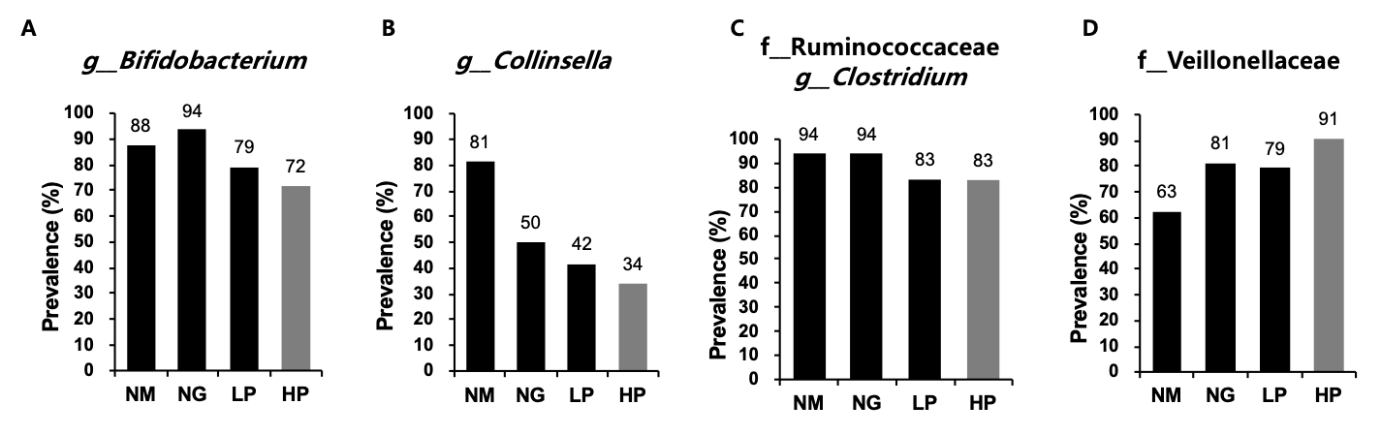


**Supplemental Figure 3.** Microbial composition at the genus level in patients with different RF levels. Prevalence of (A) *Bifidobacterium*, (B) *Collinsella*, (C) *Clostridia,* and (D) *Veillonellaceae.* Prevalence was expressed as the percentage of samples containing each bacterial genus.


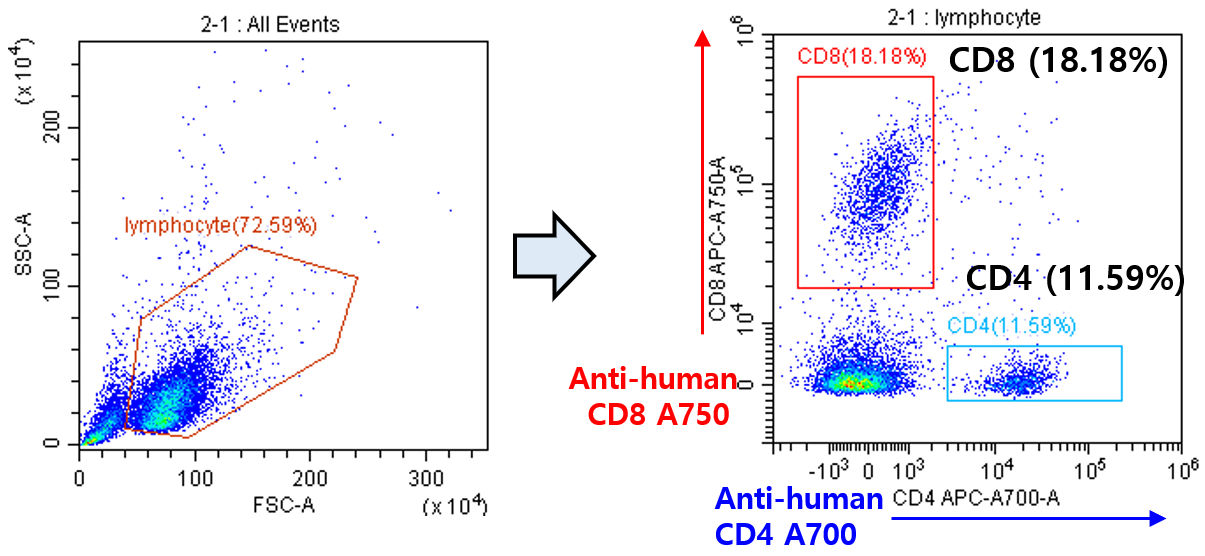


**Supplemental Figure 4.** Engraftment of RA patient PBMCs in NSG mice

To induce arthritis in humanized mice, 5x10^5^ RA patients PBMC were injected into tail vain. At 2 weeks after orbital blood was collected to confirm engraftment by flow cytometry. The engraftment of the patient PBMC was confirmed by analyzing the expression of CD4 and CD8 T cells using human antibodies.
